# What is the Point of Social Media? Corporate Purpose and Digital Democratization

**DOI:** 10.1007/s13347-025-00855-y

**Published:** 2025-02-20

**Authors:** Ugur Aytac

**Affiliations:** Department of Philosophy and Religious Studies, Utrecht University, Janskerkhof 13, Utrecht, 3512 BL The Netherlands

**Keywords:** Social media, Corporate purpose, Corporate governance, Public sphere, Digital communication

## Abstract

This paper proposes a new normative framework to think about Big Tech reform. Focusing on the case of digital communication, I argue that rethinking the corporate purpose of social media companies is a distinctive entry point to the debate on how to render the powers of tech corporations democratically legitimate. I contend that we need to strive for a reform that redefines the corporate purpose of social media companies. In this view, their purpose should be to create and maintain a free, egalitarian, and democratic public sphere rather than profit seeking. This political reform democratically contains corporate power in two ways: first, the legally enforceable fiduciary duties of corporate boards are reconceptualized in relation to democratic purposes rather than shareholder interests. Second, corporate governance structures should be redesigned to ensure that the abstract purpose is realized through representatives whose incentives align with the existence of a democratic public sphere. My argument complements radical proposals such as platform socialism by drawing a connection between democratizing social media governance and identifying the proper purpose of social media companies.

## Introduction

Political philosophers, legal scholars, and business ethicists have been arguing for some time that Big Tech corporations enjoy quasi-governmental and democratically unaccountable powers (Taylor, 2021; Sharon, 2021; Aytac, 2024a; Oldenbourg, 2024). These powers pertain to, among others, arbitrary interference with online speech (Everett, 2018; Dwyer, 2020), algorithmic curation of public opinion (Christiano, 2022), and undue influence on public policy-making authorities due to the problematic levels of reliance on Big Tech services (Sharon, 2021). There is now a wide range of proposals to make Big Tech’s techno-political powers accountable to the public, including the calls for platform socialism that ensures democratized firms and common ownership of critical digital resources (Muldoon, 2022a), data cooperatives that enable large groups of users to co-manage and coordinate their personal data (Fischli, 2024), and more extensive government regulation with the support of a network of institutions including “public watchdog organizations, parliamentary committees, national data regulators and sector-specific commissions” (Taylor, 2021, p. 916).

This paper introduces a new angle in the debate by articulating a distinct entry point into Big Tech reform. I argue that *the purpose paradigm* should be deemed an essential aspect of an adequate corporate reform in the tech industry (Mayer, 2021; Claassen, 2022).^1^ Focusing on the case of dominant social media companies and their platforms, I argue that redefining the corporate purpose of these companies is crucial to ensure that their corporate power is legitimate and promote the democratic functions of the public sphere (Balkin, 2015). Given their crucial role in the public communication infrastructure, I contend that social media companies should have *the corporate purpose of creating and maintaining a free*,* egalitarian*,* and democratic public sphere.* We need to strive for a reform that redefines these companies’ purposes and incorporates political values into them. This is important for two reasons. First, mere government regulation without transforming the internal structure of Big Tech is not able to democratically contain a large area of corporate discretion where social media companies can still exercise illegitimate political power (Langvardt, 2018, p. 352).

Second, radical proposals such as platform socialism or data cooperatives are incomplete without a well-delineated set of corporate purposes (Muldoon, 2022a; Fischli, 2024). Even when tech corporations are understood as a site of democratic contestation with multiple stakeholder groups in platform socialism, social media companies have important democratic functions that should transcend the special interests of employees, consumers or local communities.^2^Their corporate governance should be oriented towards these democratic goals in a way that goes beyond special interests. Although the way these political values are interpreted and realized will ultimately be determined by public deliberation and hegemonic struggles among different interest groups, preserving them as a distinct and explicit purpose of social media corporations is vital. An explicit purpose “binds these constituencies together” as a corporate entity with a reason for existence (Levillain & Segrestin, 2019, p. 642). This is likely to filter out the gravest forms of opportunism and rent-seeking behavior from stakeholder groups as the corporate purpose gives rise to binding fiduciary duties (Claassen, 2024).^3^ A corporate purpose derived from democratic values provides a proper framing for stakeholders’ contributions to the decision-making process, emphasizing the realization of shared values rather than the advancement of other stakeholder interests.

Introducing the purpose paradigm into Big Tech reform has important implications for what kind of corporate governance structures should be created in these companies. The basic idea is that there should be a fit between the purpose of social media companies and the kinds of actors empowered in their corporate governance structures. This argument repurposes the agency theory in the economics of the firm (Jensen & Meckling, 1976; Fama, 1980), particularly its variations that depict principal-agent relationships between stakeholder groups and corporate boards (Hill & Jones, 1992; Lan & Heracelous 2010; Sjåfjell, 2018). The agency theory suggests that corporate boards as agents are obliged to realize what they take to be the best interests of their principals. For many, shareholders are the principal as they are “risk bearers” whose financial interests are advanced by a firm’s profitability (Easterbrook & Fischel, 1996, 36–39). However, when the objective function of a corporation includes democratic purposes, particularly its transformation of the digital public sphere, the democratic citizenry will be the primary risk-bearing group, and hence quasi-principal in relation to that purpose. In a way, citizens of a polity are a proxy for the purpose of social media companies to create a democratic public sphere. This is because deviations from democratic purposes typically serve other special interest groups, whereas citizens are disproportionately worse off. As a result, the appropriate constituency that is particularly well-positioned to protect the purpose of creating a democratic public sphere is beyond conventional stakeholder groups such as employees, consumers, or local communities. The proper constituency is rather the entire democratic citizenry of a political community, which bears the risks associated with an anti-democratic public sphere. This has implications for corporate governance: the interests of this constituency should be furnished with certain privileges and represented in corporate decision-making through their own agents. In the final part of the paper, I will tentatively discuss these implications.

The paper makes two key contributions to the literature. It complements the existing democratic theory approaches to social media by linking popular control to the proper purpose of these companies. This not only gives us a reason to advocate corporate democratization but also to articulate the desirable civic mode in which any popular control should be exercised, i.e., in a way that furthers the explicit purpose of the corporation rather than special interests. Such specification is needed to ensure that the corporate governance of the digital public sphere is still agonistic and contestatory, but not reducible to partisan struggles to extract resources from the firm. Additionally, my argument recalibrates the political and economic theories of the firm to offer a novel path in the debate on democratizing Big Tech. This is a surprising conclusion given that the agency theory is often utilized to justify anti-democratic firms (Jensen & Meckling, 1983). By changing the perspective about what kind of risks matter and who bears them, I show that the same economic theories can be used to arrive at different normative conclusions.

Let me add a caveat about the goal of the paper. While any reform of purpose and governance requires amendments in corporate law, this paper does not offer a legal reasoning, articulating what specific resources are available to support such a reform in the legal terrain. My aim is more conceptual and philosophical in that I show how the purpose paradigm can provide a normative reorientation in the way we think about Big Tech reform, and why this is an improvement addressing the shortcomings of other normative frameworks. This will hopefully lay the groundwork for more concrete and legally grounded proposals, which will further test the viability of the paradigm.

The paper proceeds as follows: I start by briefly summarizing the common diagnoses and criticisms of social media companies’ political powers. I then discuss some of the available reform proposals and highlight their limitations. I explain how these limitations can be alleviated by introducing the purpose paradigm into tech industry reforms. Following this, I argue that the corporate purpose of social media companies should be redefined in line with democratic values. Lastly, I show why the transformation of corporate purpose should lead to a wider democratization of these corporations’ organizational structures with a privileged role for the representation of citizens in corporate governance.

## Social Media Companies and Their Political Powers

Dominant social media companies exercise concentrated control over democratic processes of political participation and public opinion formation.^4^ Some argue that they have the power to “directly interfere with” online speech and democratic contestation (Everett, 2018; Aytac, 2024a, 12). Although the protection of free speech and political rights are typically defined in relation to the risk of state interference, there are a number of arguments to expand its scope and treat social media companies as quasi-governmental entities. Considering the importance of major social media platforms for effective civic engagement and political communication, Aytac (2024a, 14) contends that “the costs of quitting social media are unacceptably high.” The scale and network effects of major platforms such as Facebook and YouTube implies that speech on their platforms are far more effective than other available platforms (Taylor, 2021, p. 903; Larson, 2020). While not backed by coercive power as in government censorship, the content moderation of social media companies is analogous to governmental interference due to the lack of reasonable alternatives.

Further, social media companies transform the field of political competition in ways that generate democratic legitimacy deficits, for example, through the provision of services such as microtargetting: “using algorithms to target messages to certain groups of persons who would be particularly likely to be moved by those messages given all the data collected about what they and others like them would be moved by” (Christiano, 2022, p. 113). The provision of such services without adequate accountability mechanisms implies a problematic power to transform democratic politics. For some, microtargeting risks “undermin[ing] meaningful democratic deliberation” (Harker, 2020, p. 151). Another line of criticism can be that microtargeting enables self-justifying power, empowering political elites to legitimize their social position and power by using the very same power via technology (Williams, 2005; Aytac & Rossi, 2023). In a way, social media companies help the powerful to legitimize their privileged position by relying on their purchasing power rather than genuinely appealing to the values and interests of voters. Similarly, algorithmic curation of visibility introduces a novel form of political inequality in public discourse “when social media algorithms amplify particular political messages over others in a systematic way” (Taylor, 2021, p. 899). There is mixed evidence and considerable disagreement about to what extent such curation amplifies online polarization and filter bubbles (Allcott et al., 2020; Cinelli et al., 2021; Dubois & Blank, 2018; Guess et al., 2018). However, normative complaints concerning unequal visibility seem to be independent of the accuracy of such empirical claims.

Lastly, social media companies’ interference with political speech and transformative effects on political competition implies an extremely concentrated control over the public sphere. It is already suggested that concentration in conventional media is problematic due to its reduction of diversity in public opinion formation (Hendrickx & Van Remoortere, 2024). Our reliance on social media platforms gives rise to a similarly problematic relationship: a small number of corporations determine, monitor, and enforce the norms about what speech will be permissible and visible in online venues where speech can generate significant political outcomes. Another crucial issue is that the degree of concentration in social media seems much more severe than in conventional media (Noam, 2016, 1022; Statcounter, 2022; Aytac, 2024b). As the critics noted, these companies might not be permanent monopolies as the “market leaders” in social media have been changing over the last three decades (Thierer, 2012; 274–278). However, this does not necessarily show that there are no temporary monopolies that can effectively exercise undue and concentrated control over public opinion formation at any given time.

## Some Limitations of Available Reform Proposals

Let me now discuss several reform proposals and highlight their limitations.^5^ Calling for more extensive government regulation is one dominant strand in the debate on Big Tech reform. Taylor (2021) presents a sophisticated version of this approach according to which regulatory solutions should have a structural focus, implying the creation of a regulatory ecology with a large network of governmental and non-governmental organizations. Taylor’s approach involves two steps: pressuring governments to take a more active stance in the regulation of Big Tech through the deliberative activities of “watchdog organizations” and formal bodies such as “parliamentary committees”. Then, concrete regulatory steps are to be followed in the context of national jurisdictions and international law (Ibid., 915–917).

The content of such a structural regulatory approach can be further specified depending on one’s legal reform strategy. Some favor categorizing social media platforms as a type of public utility, which would expand the scope of governmental supervision beyond corporate law. As Rahman (2018, 235–236) suggested, when a small set of companies exercise quasi-monopolistic control over crucial information provision, public utility regulation can be employed to create “new forms of *oversight* and *accountability*”, revolving around “public utility-style values such as norms of nondiscrimination, common carriage, and public safety.” Another route is to draw on anti-trust law as a tool of regulation. The proponents of this approach primarily focus on dispersing the powers of social media companies through a regulatory intervention that “aim[s] at producing smaller companies”, and “keeping new startups from being bought up early” (Balkin, 2021, p. 91). Another objective is to “separate different functions that are currently housed in the same company”, for example isolating digital communication functions from data commodification activities (Ibid., 91).

While I acknowledge the importance of these regulatory frameworks, their limitations should also be highlighted. Regardless of the specific route one opts for, regulatory approaches have difficulty in shaping corporate behavior in a large area of discretion (Langvardt, 2018, p. 352; O’Shea, 2020, p. 558). Regulatory schemes often impose side-constraints about what corporations should not do whereas social media companies can still exercise their political powers in uncharted territories until a new regulation reacts to the most recent developments (Claassen, 2024, p. 320; Aytac, 2024a, 20). More sophisticated attempts to shape these companies’ incentives through regulation often create suboptimal and unintended consequences. For example, when the requirements of regulations about content moderation are stated meticulously, companies “engage in overkill if they are focused on penalties for underenforcement”, worsening the complaints about online censorship (Langvardt, 2018, p. 352; Aytac, 2024a, 20). Further, especially in the case of public utility regulation, a “regulatory capture” is quite a likely outcome given that the existing liberal democratic political institutions are not particularly immune to corporate lobbying and other “rent-seeking” advocacy (Thierer, 2012, 271–272).^6^ The risk of regulatory capture by well-organized groups is established in the political science and public policy literature (Dal Bó, 2006). This phenomenon systematically colors the outcomes of regulatory attempts.^7^

The use of anti-trust law can be an effective means of dispersing corporate power in social media. However, some argue multiplying the number of platforms can amplify problematic enclave formation in the digital public sphere (Aytac, 2024b). Others hold that such fragmentation can be a democratically valuable form of diversity, leading to multiplicity of information communities with a variety of speech rules (Balkin, 2021, 76–78). While I acknowledge the value of having various communities in a democratic public sphere, it seems this argument overlooks the need for a shared infrastructure where these sub-communities can interact with each other, and form a broader democratic public. A democratic public sphere presupposes a shared environment as much as a plurality of speech communities (Forestal, 2021, 27–30).

Another route to Big Tech reform is to offer a more radical outlook. One such radical reformist solution is James Muldoon's (2022a) defense of platform socialism. A defining feature of Muldoon's (2022b, 7) socialist model is that it relies on “a more expansive set of participatory rights that would enable democratic collectives to control public services and play an active role in shaping the structure and role of these institutions”. In line with the proponents of public utility regulation, Muldoon's (2022a, 2022b, 18) platform socialism conceives of social media and other digital infrastructure as essential resources in need of comprehensive democratic governance. The envisaged institutional model is less bureaucratic and more participatory. Platform socialism aims to achieve an egalitarian and participatory digital order through instituting three key pillars: (i) “social ownership over digital assets”, (ii) “workplace democracy”, and (iii) “multistakeholder governance” where all affected parties participate in decision-making processes (Muldoon, 2022b, p. 8).

The main limitation is that Muldoon’s model of platform socialism does not offer any mechanism to address possible rent-seeking behavior of stakeholders. Social media platforms seem to have key democratic functions that are not necessarily reducible to narrow stakeholder interests. Following Balkin (2021, 75), we can roughly suggest that social media platforms should fulfill three democratic functions: they should (i) “facilitate public participation in art, politics, and culture”, (ii) “organize public conversation” by creating manageable user experience, and (iii) “curate public opinion” through algorithmic filtering and “community standards”. If we agree that these functions should be primarily informed by democratic values, a pure reliance on multistakeholder governance is likely to generate certain disruptions. The key stakeholders might be said to have at least some interests that are at odds with democratic values. For instance, employees might be financially better off if they prioritize creating a social media platform that maximizes its entertainment value, rather than the role of facilitating public debate due to concerns about profitability. One might reply to this objection by suggesting that all affected parties are included in multistakeholder governance, including the entire democratic citizenry. After all, citizens can be expected to counteract special interests, as I will argue in the upcoming sections. However, there is no guarantee that citizens *qua* citizens participate in multistakeholder decision-making processes. People have many different roles and identities. They are consumers, service users, partisan supporters, and citizens all at the same time. In the absence of certain norms that frame and guide corporate governance processes, individuals who are selected as citizen representatives can instead participate with their consumer preferences in mind, say disproportionately prioritizing enjoyable user experience over democratic functions. Different stakeholder groups counterbalancing each other is not a solution either. Consumers’ preferences for pleasurable leisure combined with the economic interests of employees and shareholders might generate an equilibrium that is highly at odds with the democratic functions of social media. Relying on the balance of power between different actors is not a sufficient safeguard against the risk of social media platforms being pulled in the directions of various adverse incentives.

As a result, I believe platform socialism should be supplemented with an additional mechanism of orienting corporate governance towards democratic values beyond perceived stakeholder interests. It is not that the interpretation of these democratic values can be non-partisan. I envisage a contestatory and agonistic site of politics within the corporate governance of social media (Brand et al., 2020, p. 13). Further, I acknowledge that interpretations of democratic values may be colored by competing stakeholder interests (Mouffe, 2000, p. 13). Still, incorporating these values into social media companies is likely to achieve two things. It will give social media corporations an independent reason for existence, which can filter out the worst forms of opportunistic, rent-seeking stakeholder behavior. Even if convergence on how to interpret social media’s democratic function is not possible, the existence of shared collective objectives will limit how much different groups can push for arbitrary demands. These would be obviously at odds with the company’s reason for existence. Consider a scenario where the platform regulation of fake news or online hate speech is in tension with the company’s business model. When there is such a tradeoff, an explicit corporate purpose that prioritizes truthful and democratic public communication can rule out the validity of economic considerations in the first place. This would happen even if there is some disagreement about what counts as hate speech or fake news.

As we will see in more detail in the rest of this paper, an explicit purpose would help a social media company better fulfill its political functions in public opinion formation processes. This is because any policy proposal in its corporate governance would have to be defended in a way that bolsters these democratic goals, cultivating a mechanism of accountability in relation to democratic purposes. For example, algorithmic design choices at the corporate policy-making level would have to be formulated in terms of whether they serve the socially beneficial purpose of a platform rather than harvesting as much user data as possible. In the remainder of this paper, I will explain how the purpose paradigm in corporate governance can help us address these issues.

## What is the Purpose Paradigm?

Mayer (2021, 888) contends that any adequate reform of corporate capitalism should prioritize the “question about why business exists and is created, what it does and aspires to become, namely its purpose…” The conventional answer to this question has been the idea that the primary task of business corporations is to “maximise value” for their shareholders (Jensen, 2001, p. 300). However, corporate activities have wide-ranging spillover effects on the environment, communities, and employees (British Academy, 2019, Mayer, 2021, 891–892). The proponents of the purpose paradigm suggest that introducing socially beneficial goals into corporate purpose is an effective way to ensure that these broader societal interests are taken into consideration in corporate governance (British Academy, 2019, 16). There are two key assumptions behind this claim. The first assumption is that market competition is not an adequate side-constraint for corporate activity as markets often generate socially suboptimal outcomes (Ibid., 17). Consider the example of how intense competition and pressure to maximize shareholder value can incentivize extremely risky investment behavior with adverse long-term effects on the environment (Ciepley, 2013; Claassen, 2024).

The second assumption is that government regulation systematically fails to adequately contain private corporate power, which always finds ways to “circumvent regulations and turn them to their competitive advantage using them as barriers to entry to new firms entering their markets” (Mayer, 2021, p. 891). Mayer’s purpose paradigm offers a third alternative: reshaping the nature of corporate agency so that corporate incentives and interests are defined in terms of socially beneficial objectives. Rather than shareholder value maximization as the default option, corporate activities should be oriented towards socially desirable goals that involve “produc[ing] profitable solutions to the problems of people and planet” (Mayer, 2020, p. 229). In the more radical versions, corporate purpose has lexical priority over profit or profit can be even entirely excluded from legitimate objectives (Lankoski & Smith, 2018). In this lexical priority view, while profits can be a desirable byproduct, no amount of profit suffices to sacrifice corporate purpose, defined in terms of some socially desirable objective. Many suggest that “the founder of a company” should decide what purpose their organization will serve, and “what problems the company is solving”, unless corporations “perform important social functions” (British Academy, 2019, 16; Mayer, 2020, p. 230). According to the proponents of this view, the emergence of a corporate purpose is supposed to steer corporate activity in a socially and environmentally responsible direction to the extent that corporate boards are legally and ethically obliged to realize their declared purposes.

Further clarification is needed on the relationship between corporate purpose and corporate governance. Some present the purpose paradigm as an alternative to the debates focused on how decision-making authority should be allocated between corporate stakeholders (Levillain & Segrestin, 2019, p. 642). I believe this idea has shortcomings, and corporate purpose should go hand in hand with corporate governance reform. First, the status quo that gives shareholders disproportionate powers, e.g., right to elect directors, already undermines the idea that corporate purpose can be realized in ways that genuinely transcend narrow group interests (Strine, 2017; Davis, 2021). It also seems arbitrary that the original founders or shareholders are authorized to determine corporate purpose unilaterally. If corporate purpose is defined beyond wealth creation, the economic arguments for shareholder primacy lose their force (Jensen, 2001). An argument from private property rights is insufficient to establish shareholder primacy either: shareholders do not enjoy ownership “rights over corporate assets” in any straightforward sense due to “asset lock-in” (Ciepley, 2013, 143–144, 146). Once a corporation substantially grows and significantly impacts the legitimate interests of other groups, it is not clear why these groups should be excluded from procedures to initiate a revision in corporate purpose. Their disenfranchisement would resemble depriving future generations of the right to change a constitution their ancestors ratified.

Lastly, even if it were possible to create a genuinely independent corporate board that can stay committed to declared corporate purpose, it would be undesirable. Mayer (2021, 889–890) suggests that corporate purposes should be relatively well-delineated and oriented towards concrete outcomes. However, they can still involve normative tradeoffs in decisions about how to achieve those purposes. Mayer (2021, 890) discusses the endorsement of a corporate purpose with the example of a pharmaceutical company, Novo Nordisk, which gradually expanded its main objective from “produc[ing] insulin” to “help[ing] people treat Type 2 diabetes” in any way possible. Although this can be construed as a specific goal, it is still sufficiently vague to be normatively contestable. For example, how should Novo Nordisk decide when it faces a choice between a high-risk investment for a highly effective drug and a low-risk investment for a drug delivering only moderate improvement in life expectancy? Or should the company maximize its diabetes treatment efforts by introducing slightly more exploitative working conditions? Technocratic and independent corporate boards are not necessarily more skilled or entitled to resolve such normative tradeoffs than thousands of patients whose lives depend on these decisions or employees who can be adversely affected by the way purpose is interpreted and realized. If the purpose was normatively specific and incontestable, it would perhaps be possible to legitimize independent boards as a means of protecting corporate purpose from adverse incentives. However, the purpose itself is often subject to legitimate political contestations about how to specify and implement it (Brand et al., 2020, p. 13). Hence, corporate purpose should be supplemented with proper mechanisms of democratic accountability so that corporate power can be responsive to the affected parties’ legitimate interests and viewpoints. There is still normative vagueness about purpose despite democratic contestation. But at least, the possibility of holding a board accountable to other parties makes the board’s way of dealing with vagueness more legitimate. 

Let me note that this democratic twist doesn’t make the purpose paradigm entirely reducible to multistakeholder governance (Pek, 2023, p. 262). One stakeholder group can still be identified as the guardian of purpose if their incentives are relatively aligned with it. This also implies granting that group certain privileges in the governance structure despite the broader framework of multistakeholder governance (Levillain & Segrestin, 2019, p. 639). Additionally, corporate purpose creates a filtering mechanism about what kind of inputs are deemed legitimate in democratic decision-making processes. Just like there are constraints and guidelines about how to participate in state politics, e.g., staying committed to your party programme or no incitement to violence, corporate purpose provides a civic framework about how stakeholders should steer the governance of the corporation in terms of what kind of interests they are expected to pursue. This is primarily supposed to minimize the influence of rent-seeking and private interests that would undermine the integrity of the corporation. In the next section, I apply the purpose paradigm to social media companies. But let me first address one final issue in the following paragraph.

Regulatory capture is also a problem for the purpose paradigm as revising corporate purpose would require some legislative changes. However, a purpose reform still bears advantages compared to regulatory approaches. In both cases, there needs to be a suitable moment for a progressive legislative change. Exogenous factors such as economic crises or corruption scandals often create a “window of opportunity” to initiate a radical reform against entrenched interests (Brückner & Ciccone, 2011; Doeser and Eidenfalk, 2013; Birkmann et al., 2010). Given the level of lobbying activity and entrenched interests in the corporate economy, it is reasonable to believe that an appropriate window of opportunity is needed, ensuring that there will be enough public support for reform and the alignment of political elites’ will with the public. The problem is windows of opportunity are temporary. The public’s attention does not stay focused as the salience of a policy issue dissipates. This means entrenched corporate interests can mobilize to undo previous achievements. Political scientists call this problem “policy drift”, which is defined as “the transformation of a policy’s outcome due to the failure to update its rules or structures to reflect changing circumstances” (Galvin & Hacker, 2020, p. 216).

The purpose reform is likely to perform better than conventional regulatory approaches in the face of policy drift problems. In regulation, the efficacy of solutions depends on the enforcement of existing rules. Once the window of opportunity is over and the rules cannot be adequately updated, this will be a major setback for policy goals. Through a purpose reform, the locus of decision-making is being shifted to the corporation’s own agency without the need for continuous regulatory intervention from outside. Once the window of opportunity is over, the corporation would continue to pursue its new goals thanks to its reformed decision-making structure. 

## Social Media Companies and Corporate Purpose

### What Should be the Purpose of Social Media Companies?

Let me start by discussing why social media companies should have a socially beneficial purpose in the first place. Remember the democratic functions of social media Balkin (2021, 75) identifies: these platforms should (i) “facilitate public participation in art, politics, and culture”, (ii) “organize public conversation” by creating manageable user experience, and (iii) “curate public opinion” through algorithmic filtering and “community standards”. Social media platforms’ curation of public opinion ranges from politics to arts and culture, as well as different life style expressions. Such organization of public opinion determines what issues and ideas will be visible to citizens, and ultimately shapes the democratic life and political culture. There is a sense in which this is a form of political power.

Keep in mind that the digital public sphere is a necessarily governed site of public communication. Without substantial curation, moderation, and information processing, these platforms would simply fail to fulfill their communicative functions. It is not an option to have a non-governed digital public sphere unless we are willing to embrace informational cacophony. As a result, social media platforms’ organization of the digital public sphere is a given in our contemporary political context. They fulfill important public functions. The question is whether we should let certain institutions fulfill public functions without them having public purposes. There are strong reasons to believe that “public functions” should be fulfilled by institutions with public values (Cordelli, 2020, 7). Without a public purpose, it is more difficult to hold these institutions accountable for the exercise of their powers (*Ibid.*, 193). Public purpose attribution sets a standard by which the institution can be assessed and held accountable. In contrast, private purpose allows these companies to fulfill public functions with private interests in mind (*Ibid.*, 220). In such cases, any corporate conduct would be permissible through the standards of profit-making insofar as they obey the law.

Further, it is not a category mistake to attribute public values to private entities. Ciepley (2013, 148, 154) carefully shows that the business corporation and its legal privileges (e.g., asset lock-in, limited liability, entity shielding) are “creations of government” and concessions from the state in return of “promised public benefits”. This means that the purpose of these organizations is not set in stone. In contrast, the purpose of corporations is a function of their presumed utility to the public. The profit-driven purpose of business corporations is recognized in virtue of their productivity- and growth-promoting economic effects (*Ibid.*, 143). Historical evidence is abundant with examples of governments that granted permissions to incorporate and “reserved the right to rescind or revise” those permissions (*Ibid.*, 139). Ultimately, incorporation is a legal construct that can be revised due to alternative considerations of public interest. 

I will now articulate a potential corporate purpose for social media companies and explain why this is a reasonable alternative. Keep in mind that this argument applies to dominant social media platforms as smaller companies are not able to wield political powers mentioned previously. Balkin (2021, 75) discussion of social media’s democratic functions is a good starting point. If one considers the critical diagnosis of social media companies’ political powers, discussed in the first section, one can see that the problems are primarily about disruptions in these democratic functions. For example, the power of social media companies to interfere with online speech determines the terms of “public participation” in cultural and political life (*Ibid.*, 75). Similarly, algorithmic filtering and microtargeting “curate public opinion” in normatively objectionable ways (*Ibid.*, 75): they make public opinion formation disproportionately responsive to the preferences of corporate service providers and wealthy political elites that purchase these services (Christiano, 2022, p. 122).

I believe addressing these problems concerning the three functions of social media requires a democratic corporate purpose (when they reach a certain size that makes them significant for the public sphere): *social media companies should be obliged to create and maintain a free*,* egalitarian*,* and democratic public sphere.* Call this *the democratic purpose*. There are three reasons why the democratic purpose is suitable for the corporate purpose of social media companies. First, our abovementioned diagnoses largely stem from the rise of concentrated and unaccountable powers in the domain of digital communication with significant effects on opinion formation, civic engagement, and political competition in the public sphere (Simons & Ghosh, 2020; Aytac, 2024b). These processes and practices are all related to the functioning of the public sphere understood as a domain of democratic influence where citizens impact political life collectively through participating in public discourse. Hence, counteracting concentrated and unaccountable powers would amount to creating a properly democratic public sphere. There are different democratic theories each having a distinct way of articulating what this democratic influence should look like. Some emphasize “egalitarian public deliberation” (Habermas, 1996; Christiano, 2008, 197–201), whereas others understand it as a way of “organiz[ing] the collective power of” ordinary citizens to minimize oligarchy or domination (Pettit, 2013; Klein, 2022, p. 39). One common feature is that our grievances, as well as a variety of democratic theories, are informed by a deep commitment to the idea of a public sphere that is more democratic and egalitarian than the current social media landscape marked by concentrated and unaccountable power.

Addressing these problems through the purpose paradigm requires a democratic purpose, obliging social media companies to alleviate power and status asymmetries in the digital public sphere. This would have important implications: the governance of digital information flows and online speech would have to be made more *egalitarian*: either by limiting concentrated corporate power or by making corporate power democratically inclusive. Further, social media companies would be obliged to counteract the existing structural patterns of *inequality* and censorship in the wider public sphere through their content moderation and algorithmic curation practices, for instance, by empowering marginalized communities to speak up without the fear of hostile reactions (Patberg, 2025).

Second, the democratic purpose is broad enough to be normatively contestable and substantive enough to steer the corporate governance of social media platforms towards some clear goals. Let me explain. The proponents of the purpose paradigm often suggest that “a [good] purpose is precise about what problems it is seeking to solve…” (Mayer, 2021, p. 890). However, this is particularly problematic when corporations such as social media companies have political functions. Political values are often “essentially contested” due to their “internally complex” structure (Gallie, 1956, 171). For instance, when the task is to create a democratic public sphere, this involves many different components as the normative idea of a public sphere can have various functions such as “the inclusion of multiple and plural voices”, generating epistemically healthy “preferences, opinions, and decisions”, and “promot[ing] mutual respect among citizens” (Mansbridge et al., 2012, 11–12). As a result, the purpose should be sufficiently broad otherwise it might not be able to do justice to the diversity of democratic values and concepts. For example, there might be inevitable tradeoffs between ensuring accountability in online speech communities through identity verification and increasing democratic participation resulting from the relative safety of anonymous accounts (Asenbaum, 2018, 463–464; O’Neill, 2022). The democratic purpose should be “internally complex” to account for such tradeoffs (Gallie, 1956, 171).

A narrow purpose is also likely to generate a legitimacy deficit. Different group interests can be mapped onto different conceptions of democracy, each prioritizing some components over others. For instance, marginalized communities might be silenced by an excessive focus on promoting online civility because such norms sometimes implicitly reflect arbitrary preferences of the powerful (Young, 2001, p. 671). If a corporate board is obliged to follow a narrow purpose, the exclusion of other dimensions or interpretations of democracy will further marginalize the legitimate interests of some groups. The democratic purpose I propose is sufficiently broad as it does not exclude any specific conception of democracy or arbitrarily prioritize one component of democracy over another.

On the other hand, the purpose should not be too broad. Otherwise, it cannot be effective in shaping the processes of corporate governance. I believe the democratic purpose provides an important orientation to shape the governance of social media companies. For example, it clearly rules out a laissez-faire conception of the public sphere as the purpose pertains to the task of creating a desirable public sphere. It presupposes an actively governed conception of digital communication. An actively governed conception of the digital public sphere stands in the way of social media companies evading responsibility, which was what happened in the case of widespread “disinformation campaigns” on Facebook (McCarty, 2022). Similarly, the proposed purpose opens up the possibility of addressing substantive inequalities in the public sphere because it is not limited to promoting free speech and formal equality. Lastly, as the purpose explicitly emphasizes the democratic character of the public sphere, it counterbalances individualist and depoliticized interpretations of freedom and equality, e.g., consumer freedom to enjoy social media platforms.

Third, the democratic purpose can also accommodate the risk of overpoliticization, or strike a balance between the risks of excessive politicization and excessive depoliticization (Aytac, 2021). Although social media platforms are significant for democratic public debate and opinion formation, they are not all about politics. They also operate as a domain of experimentation that hosts various aesthetic, ethical, and evaluative expressions concerning individual lifestyles and subcultures (Balkin, 2021, p. 77). Such non-political uses of social media have a collective dimension as they cluster “around common topics of interest” (Bruns & Highfield, 2015, p. 65). However, they can still be seen as a source of individuality and heterogeneity in public life. First, the visibility of and interactions between different clusters expose users to a wide range of lifestyle expressions, which can generate unique combinations for each and every user. This is especially the case when “temporary issue publics”, such as Twitter hashtags, bring together a diverse group of users (Ibid., 67–70). Such an environment of diversity can only be secured by a strong presence of various cultural uses of social media. This is beneficial to promote pluralist societies where a wide range of experimentation about different conceptions of the good life is possible. In contrast, reducing the democratic purpose to political functions would impose on citizens a particular conception of the good life according to which civic participation is central. Hence, the governance of social media should not be overpoliticized to such an extent that its political functions crowd out cultural functions.

The democratic purpose can accommodate such value pluralism depending on one’s interpretation of a free, egalitarian and democratic public sphere. One advantage is that freedom and equality are two distinct components of this purpose, and this creates the conceptual space in which freedom can mean something other than equal political status or participation in contrast to certain republican conceptions of freedom (Arendt, 1960). Such a differentiation between freedom and equality enables the democratic purpose to support freedom as an opportunity-concept, expanding individuals’ choice menu regarding the lives they might want to live beyond politics and cultural experiments they might want to conduct in digital public life.

One might still hold that the democratic purpose does not sufficiently specify what is meant by freedom, equality, and democracy, and hence can’t offer useful guidance. Relative indeterminacy of these values is a feature rather than a bug of my proposal. The democratic purpose is analogous to what Mouffe (2000, 13, 29) calls “the common symbolic space” in liberal democratic orders: a minimal yet foundational normative common ground that binds various actors together in a community despite the ineliminable conflicts about “the correct and definite interpretations” of these values. Mouffe suggests (2000, 113) “liberty and equality” are the basic and shared normative essence of liberal democracies although partisan conflicts about the proper specification of such values are not likely to be determined rationally. The minimal and vague content of the democratic purpose similarly presupposes that values associated with the digital public sphere are bound to generate political conflicts with no resolution. That’s also part of the reason why corporate purpose should be realized through an adequate governance procedure tailored to it. Only by establishing a political procedure that addresses power imbalances and adverse incentives of actors, can we reasonably hope that an acceptable interpretation of the democratic purpose will emanate from processes of contestation and deliberation. This is also where I diverge from Mouffe’s agonism. Although I agree that the interpretation of a value will always be colored by hegemonic struggles, there is a sense in which certain actors’ interpretations are closer to the shared common ground and hence less distorted due to the relative absence of adverse incentives, which I will further discuss in the next section.

Further, I need to discuss the relationship between the democratic purpose and profit objective. Clearly, there are alternative ways of combining financial and political goals in the objective function of social media companies. For instance, one might ask why I do not conceptualize the requirements of a democratic public sphere in terms of side-constraints that would determine the limits of profit-seeking (Claassen, 2024, p. 320). Insofar as the democratic quality of a public sphere remains above a certain threshold, corporate purpose might still be profit or shareholder value maximization without undermining our political values (Lankoski & Smith, 2018, p. 250). However, there are several problems with this moderated version of the purpose approach. I believe their line of reasoning is plausible if we can straightforwardly quantify the democratic qualities of a public sphere. In that case, profit maximization can be the guiding principle insofar as it does not drive down democratic qualities below a threshold value. However, what counts as a free, egalitarian, and democratic public sphere involves many contextual normative assessments that are not possible to quantify. For instance, a generic definition of online hate speech cannot be adequate to govern the digital public sphere. Context-specific judgments will be required in many cases of governing online speech. The problem is that quantification requires working with aggregates that erase contextual factors and nuances. In the absence of a plausible way of quantifying normative values, the risk is that profit maximization will unduly override the commitment to democratic values. This risk can be alleviated if profits are only instrumentally valued due to their possible contribution to the democratic purpose.

One might suggest that it is not necessary to quantify democratic demands. Perhaps, it is possible to formulate them as qualitative boundaries that need to be respected, as in Claassen’s formulation of “the duty not to dominate” in corporate purpose (Claassen, 2024, 318). However, the problem is that interpretative battles about qualitative side-constraints are more bias-prone than those about democratic demands understood in terms of the ultimate purpose. Showing that a corporate policy is merely *compatible with* democratic values is remarkably easier than establishing that it is *required by* democratic values. The higher burden of proof in the latter can pose a serious limit on how much different actors can push for their arbitrary demands induced by their “motivated reasoning” (Kahan, 2013). As the cognitive complexity of a discursive task increases, there will be a greater range of opportunities for other actors to contest and expose rhetorical moves and biases.

There is also an epistemic problem with the abovementioned side-constraint approach. There are currently unknown ways social media technologies can enhance democratic practices, be it new design features or implementation of new technologies within the same platform architecture. And these possibilities might influence the public’s preferences about what sort of democratic practices are desirable in the digital public sphere, which further affects where the threshold level should lie. If the democratic purpose is not in the driving seat and corporate boards are only concerned with it until reaching a certain minimum level, these possibilities are likely to remain dormant. As the public will be unaware of such options, the demands for improvements will not likely be reflected in the threshold level. As a result, detaching democratic values from corporate purpose would create a status quo bias, standing in the way of inventing new functions and design features that can further enhance democratic practices. This is mainly because corporate boards will not be incentivized to innovate to strengthen democratic practices. For these reasons, I believe the democratic purpose should be corporate purpose, and the commercial activities of social media companies should be assigned instrumental value. In this picture, profit is only valuable to social media corporations as it helps them realize their purpose better.

I do not claim that the democratic purpose is the only reasonable alternative. The purpose that we attribute to social media companies should be democratically mandated through legislative changes, and these decisions should be informed by public debate and consultation with various stakeholder groups. In this sense, my articulation of the democratic purpose should be seen as a contribution to broader processes of public debate rather than an ultimate verdict. Still, I believe any reasonable candidate must satisfy a similar set of desiderata: addressing the problem of concentrated power in the digital public sphere, formulating a broad but practically relevant purpose, and avoiding the risk of overpoliticization.

### Implications for Corporate Governance

Incorporating the democratic purpose into social media companies has three important governance implications. First, it fundamentally transforms the fiduciary duties of corporate boards.^8^ A corporate board “has duties of loyalty and care, so as not to betray the trust of her beneficiary” (Claassen, 2024, p. 324). Conventionally, corporate boards are thought to “owe fiduciary duties to shareholders” on the grounds that the latter are “residual claimants” in corporations (Macey, 2014, p. 333). The corporate purpose derived from a fiduciary relationship involves “promot[ing] the interests of shareholders in profit maximization” (Ibid., 343). These fiduciary duties are enforceable in various ways: shareholders “can sue to enforce the legal duties owed by directors and managers under the statute” (Strine, 2017, p. 178). They can also elect corporate boards in many corporate law systems. One major implication of the democratic purpose is to shift the focus of fiduciary duties from shareholders to “the corporation itself” (Levillain & Segrestin, 2019, p. 639). According to this view, the corporation itself understood in terms of its purpose is the ultimate beneficiary to which corporate boards are accountable. For example, when there is a tension between promoting viewpoint diversity on social media communication and maximizing algorithmically mediated selective exposure due to profit motives, the democratic purpose allows corporate boards to override shareholders’ financial interests. This is because redefining the ultimate corporate purpose of social media companies in line with democratic values transforms fiduciary relationships in the corporation.

Second, the democratic purpose requires a redistribution of power and authority within social media companies.^9^ Even when the fiduciary duties of corporate boards are redefined, a purpose reform would be toothless without a broader power redistribution (Davis, 2021). For instance, Davis suggests, if shareholders continue to elect the members of corporate boards and exercise other disciplinary powers, profit motives are very likely to override the formal purpose of the corporation. Therefore, it is essential to rethink other models of governance in which shareholders cannot dominate. Further, the corporate governance structure of social media companies should be altered to ensure that there are some groups who are incentivized and able to enforce the democratic purpose. When it is only on paper, the purpose is not going to enforce itself. One should identify and empower a relevant stakeholder group whose interests are relatively aligned with the democratic purpose. Only by doing so, can we hope that the democratic purpose has a reasonable chance of effectively steering corporate governance.

I believe the democratic citizenry of a polity is a particularly suitable group for this purpose. Citizens have an interest in realizing the democratic purpose in social media companies to the extent that they benefit from a well-functioning public sphere. When disinformation on social media is pervasive, citizens cannot make “informed” political choices (Brown, 2023, p. 48). When malicious groups game algorithmic curation, citizens’ attention is unduly controlled by manipulative actors (Karppi, 2013, p. 288). When microtargeting becomes prevalent, the existing “asymmetries of informational power” between citizens and wealthy political elites deteriorate (Christiano, 2022, p. 122). When corporate elites moderate content, citizens’ online speech is regulated by an alien power that is not democratically mandated (Aytac, 2024a). All of these attribute to citizens qua citizens a significant interest in a properly democratic public sphere.

In contrast, other stakeholder groups have additional interests likely to clash with the democratic purpose. For example, shareholders and employees have financial incentives to prioritize a business model that works for them. Governments might be tempted by the surveillance capacity of these technologies, which would boost their control over large populations (Stahl, 2016). Citizens, on the other hand, benefit from social media platforms primarily as a domain of public communication, civic engagement, and leisure time activity. In a way, they are the group that would be disproportionately harmed due to deviations from the democratic purpose. Other stakeholders might still navigate and advance their interests but citizens have much less opportunities of this kind. This makes citizens a good candidate for the enforcer role in realizing the democratic purpose. They are well-positioned and properly incentivized to prioritize socially beneficial functions of social media over its financial benefits or its use as a tool of manipulation and surveillance.

One might object that empowering citizens is hard to square with my previous objection to multistakeholder governance. I claimed that incorporating citizens into the governance of social media companies is inadequate because they might prioritize their private, consumer preferences over the political preferences they have as citizens. Let me note that the expected outcomes of authorizing citizen bodies in corporate governance vary depending on the broader institutional context. When the corporate purpose is entirely up to stakeholders’ perceived interests, we have good reasons to believe that empowering citizens will not be sufficient. However, it is a completely different task to find out which stakeholder group is relatively well-positioned to monitor and enforce a preexisting corporate purpose. Here, we can identify the democratic citizenry as the most plausible candidate as their interests aligned better with the democratic purpose than other groups. Of course, this does not guarantee that the democratic purpose will be naturally realized through citizen bodies’ self-interested behavior. There needs to be additional safeguards. One such mechanism is that citizen bodies’ obligations and activities should be legally and discursively framed around the task of realizing the democratic purpose of a social media company (through further specifications in laws and guidelines that are beyond the scope of this paper). Further, when these activities are translated into binding obligations, judicial review should also be considered as an additional disciplinary mechanism to incentivize citizen bodies not to prioritize their private consumer preferences, whether through state-level or corporate courts (Blanc, 2023).

This line of reasoning allows us to repurpose the agency theory of corporate governance, providing a solid framework to rethink the governance of social media companies. Typically, agency theorists argue that shareholders have a privileged status in corporate governance as they are “risk bearers” who should “get a residual claim to profit” (Easterbrook & Fischel, 1996, p. 36; Fama, 1980, 294–295). This is because other parties in the firm enjoy guaranteed financial returns that minimize the risks concerning the firm’s financial volatility, e.g., wage laborers’ or suppliers’ compensation are protected by contracts (Macey, 2014, 333). In contrast, the agency theorists hold, shareholders are the group that receives residual revenues whose size will be uncertain and decided by the firm’s performance (*Ibid*., 337). For this reason, they argue that realizing shareholder interests should be deemed the corporation’s purpose. This is because their incentives lead to an economy where high-performing firms ensure “that social welfare is maximized” (Jensen, 2001, p. 11). As they are residual claimants, “shareholders are incentivized to monitor” corporate boards, and sanction unproductivity and shirking (Jabotinsky & Siems, 2018, p. 357; Alchian & Demsetz, 1972), which would accelerate wealth creation in the economy. This line of reasoning also explains the agency theorists’ commitment to empower shareholders in corporate governance structures.

Inspired by the adaptations of agency theory for stakeholder interests (Hill & Jones, 1992, p. 134; Sjåfjell, 2018), one can repurpose this argument to make sense of citizens’ special role in the corporate governance of social media companies. This idea draws on Sjåfjell (2018, 115) who contends that affected communities should be deemed a principle in corporate governance. In one sense, citizens are the primary *political* risk-bearing group in relation to the democratic purpose of a social media company as they are more likely to be disproportionately harmed by deviations from such a purpose. Unlike other groups, they do not have access to mechanisms of financial reward that can generate adverse incentives incompatible with the democratic purpose. Further, citizens’ interest in a democratic public sphere cannot be protected by contracts as in protecting a supplier’s interest through a fixed-rate sales contract (Easterbrook & Fischel, 1996, p. 36). Their political interests are non-pecuniary, and there seems to be no precisely measurable metric of a democratic public sphere that can spell out citizen interests against future risks in a contract. For example, it is not clear what viable metric can be employed to measure interests like protection from online manipulation. Moreover, the normative and broad nature of the democratic purpose would create “incomplete contracts” which gives corporate boards substantial discretion in the implementation of the terms of the contract (Hart, 2017, 1732). If a contract says online hate speech should not be allowed on a social media platform, corporate boards and their proxies will still choose a specific interpretation of what counts as hate speech. Given that citizens’ political interests are in line with the democratic purpose, that they have incentives to promote the purpose, and that these interests cannot be adequately protected by contractual relations, they should be identified as a quasi-principal group in social media companies. While the actual “principal” will be “the corporation itself” understood in terms of its “purpose” (Sjåfjell, 2018, p. 114, 120), citizens should be empowered to act as the proxies of the corporation in order to effectively realize the purpose.^10^ Citizen bodies should represent the real principal, and hold the agents, i.e., corporate boards, accountable.

How can we institutionalize the representation of citizens in social media companies? I am generally sympathetic to Muldoon’s (2022a) multistakeholder democratic governance scheme if we modify it. It would be plausible to incorporate a citizen body into such corporate governance structures while maintaining their privileged position due to their quasi-principal status. Providing a blueprint of what this would look like is beyond the scope of the paper, but one can point towards some possibilities and desiderata. For instance, citizen representatives can enjoy the exclusive right to sue corporate boards due to alleged deviations from corporate purpose. One might also consider granting citizen representatives the right to appoint more corporate board members than other stakeholder groups.

Another important question is how these citizen bodies should be selected. Due to space constraints, I am unable to articulate and defend a specific model in detail. However, let me note that there are some promising ideas about instituting randomly selected citizen assemblies furnished with the authority to supervise corporate boards’ activities (Bennett & Claassen, 2022, p. 159). Others defended such bodies of ordinary citizens in the context of Big Tech corporations (Carugati & Levi, 2021, 53–57; Simons & Ghosh, 2020; Aytac, 2024b). This kind of citizen representation has a distinctive benefit compared to electoral representation, which can introduce another layer of power and status asymmetries due to its competitive logic (Aytac, 2024b). There is a sense in which citizens’ democratic interests should also be protected from electoral political elites, especially when the task is to govern the digital public sphere. This is because the democratic function of the public sphere is to ensure an accountability link between ordinary citizens and those who rule over them (Chambers & Kopstein, 2023, p. 226). Hence, it would be more prudent if we choose a selection mechanism that guarantees that various elite groups are not overrepresented in the formation of citizen bodies. Further, randomly selected citizen assemblies can counterbalance the disproportionate influence of corporate interest groups in conventional policy-making processes. While career politicians tend to be responsive to corporate lobbying, the members of randomly selected citizen bodies do not have much to gain from making concessions to organized corporate interests (Bagg, 2024, p. 98). 

Lastly, the democratic purpose is likely to transform the dominant mode of civic engagement in the corporate governance of social media companies. Such a corporate purpose provides an explicitly endorsed discursive framing about which interests and goals have lexical priority over others. For instance, no amount of profit gains can legitimize a substantial deterioration in the democratic quality of a social media platform. Say there is a choice to be made between more strictly regulating hate speech and the number of platform users. When profit maximization is the corporate purpose, a social media company might be more reluctant to sanction a large number of hate speech perpetrators unless this can be shown to be profitable for their business. However, as the democratic purpose gives rise to legally enforceable fiduciary duties for corporate boards, it is likely to filter out rent-seeking stakeholder behavior including the financial demands of creditors and employees. Each stakeholder would have to show that they advance their interests in a way that serves the democratic purpose, and corporate boards should justify their actions on these grounds. One shouldn’t be too naive about the effects of these discursive framings as competing interpretations of the democratic purpose will undoubtedly be colored by different stakeholder interests. However, it can at least filter out the most problematic forms of narrow group interests as the most blatant misrepresentations will attract stronger criticisms, and citizen bodies can always trigger judicial review when corporate boards abuse the plurality of interpretations about purpose. Since there is room for multistakeholder contestation in my model and because corporate policies should be officially justified in relation to the democratic purpose, there are discursive/cultural constraints on how much a stakeholder group can push for demands that are detached from the purpose. 

As previously suggested, there is no guarantee that citizen representatives will display civic virtue. However, some measures can be taken to minimize risks and support a civic discursive culture in corporate governance. First, citizen assemblies, when designed as deliberative mini-publics, have a number of procedural rules for their internal functioning such as, the duty to make a sincere attempt to present a good argument and justify one’s position through considerations of public interest (Grönlund et al., 2010, p. 97). The empirical findings suggest that well-structured procedures can cultivate good deliberative behavior in participants (*Ibid.*, 95). Similarly, the design of citizen assemblies can instill incentive structures to motivate participants to base their discourses on the common good understood in terms of the company’s democratic purpose rather than consumerist preferences. Afsahi (2022, 702) shows that several “facilitation techniques” and procedural norms can incentivize participants of minipublics to display good deliberative behavior. It is reasonable to hope that the internal design of deliberative spaces will encourage some degree of commitment to the purpose. 

The lexical priority of the democratic purpose over commercial interests also induces a fundamental shift in how these companies pool capital for their investment. As the endless pursuit of profits is off the table, social media companies are likely to exit shareholder capitalism in this framework. Hence, any reform in the direction of the democratic purpose should also be supplemented with additional arrangements to ease these corporations’ access to credit markets as an alternative source of capital, e.g., interest rate subsidies. Credit markets can still support innovation. Especially when the “long-term bank loan market” expands, credit markets accelerate innovation (Xin et al., 2017, p. 268). Stock and credit markets have varying effects on investment depending on the kind of innovation, as shown in Wang’s (2023, 103) empirical analysis: “credit market development promotes incremental innovation, especially in mature and large firms, but has negative impact on substantive innovation.” Such findings are particularly interesting for major social media companies, which are large corporations that can disproportionately benefit from further reliance on credit markets. 

Incorporating the democratic purpose into the governance of social media companies complements the existing reform proposals. It complements conventional regulatory approaches in that it democratically contains a relatively large area of corporate discretion by transforming the internal structure and purpose of social media companies. Although regulatory measures can still impose certain side-constraints about the basic rules social media platforms should comply with, they would be insufficient to proactively steer the direction of corporate governance. We can now envision a division of labor between regulatory attempts ensuring the liability of social media companies in relation to the violations of rules and the purpose paradigm’s more substantive prescriptions about how social media platforms should be governed.

Similarly, the democratic purpose strengthens radical democratic aspirations about Big Tech reform such as the call for platform socialism. In a way, my argument strikes a balance between procedural and substantive aspects of democratization. While the aspiration for multistakeholder democratic control is valuable, the substantive content of these companies’ corporate policies is equally important as they affect the broader political processes in the public sphere. That’s why the participatory procedures of corporate decision-making should be located within an institutional framework that states the primary goal of a social media company in line with democratic values. This will ideally maintain a compromise between democratic governance and governance for democracy to the extent that the democratic purpose and its proxies such as citizen representatives steer the participatory processes towards democratically valuable objectives.

## Conclusion

Throughout this paper I have argued that the purpose paradigm can significantly contribute to the debates on social media reform. Having identified the shortcomings of available reform proposals, I contended that we should partly focus our efforts on redefining the corporate purpose of these companies. They should have a democratic purpose: creating a free, egalitarian, and democratic public sphere. I defended the plausibility of this purpose for social media companies on the grounds that it addresses the problem of concentrated power in the public sphere, gives corporations a substantive yet not overspecified objective, and accommodates the risk of excessive politicization of social media. Lastly, I showed that the democratic purpose requires a radical transformation in the corporate governance structure of social media. This includes the reconceptualization of fiduciary duties, redistribution of power and authorities within the corporation, and transformation of civic-discursive framing of stakeholder participation in corporate governance. Most notably, such a reform implies citizen empowerment as a distinct and privileged stakeholder that is obliged to act as the proxy of the democratic purpose.

## Data Availability

Not applicable.

## Footnotes

1 I borrow from Bennett and Claassen (2022, 149–150) and Claassen (2022) the idea that the literature on corporate purpose constitutes a distinct paradigm in the debates on corporate governance.

2 See Levellain & Segrestin (2019) for a general defense of corporate purpose that goes beyond the question of who should govern the corporation.

3 By rent-seeking behavior, I refer to stakeholders’ extraction of value from the firm at the cost of the firm’s production of goods that benefit everyone involved.

4 The literature review in this section draws on my previous research (Aytac, 2024a, b).

5 I leave out the discussion of other policy proposals such as Capasso & Umbrello’s (2023) model of “social license to operate” because they focus on self-regulation.

6 The objections in this and following paragraph draws on my previous research (Aytac, 2024b).

7 Regulations might be a better alternative when a policy domain pertains to the risk of harming others, which can be construed in the form of prohibitions. In contrast, corporate purpose can play a greater role in policy-making areas where corporations are expected to discharge certain obligations even when they do the bare minimum in terms of not violating any prohibitions.

8 My analysis differs from Balkin’s (2015) conception of “information fiduciaries”. My argument appeals to a different type of considerations in grounding social media companies’ fiduciaries duties towards citizens, i.e., the corporations’ power over public discourse and political processes rather than their control over “sensitive personal information” (Ibid., 1207).

9 Some proponents of the purpose paradigm are skeptical of such proposals (Mayer, 2016, p. 62). I already discussed why the proposal of independent corporate boards is undesirable in Sect. 3. Further discussion is beyond the scope of this paper.

10 Hill and Jones (1992, 134) are skeptical about seeing “stakeholders” as “principles” although they agree that there is an agency relationship between stakeholder interests and managers.
